# Research on the influencing factors of sustainable fundraising in internet medical crowdfunding: based on dramaturgical theory

**DOI:** 10.3389/fpubh.2025.1667554

**Published:** 2026-01-09

**Authors:** Zihan Huang, Zhenyan Han

**Affiliations:** School of Public Administration, Hohai University, Nanjing, China

**Keywords:** dramaturgical theory, medical crowdfunding, charitable organizations, fundraising performance, influencing factors, sustainable development

## Abstract

Amid global population aging and escalating healthcare costs, technology-enabled medical crowdfunding has emerged as a means to improve access to care. Nevertheless, concerns persist regarding its fairness, accountability, and long-term sustainability. Grounded in dramaturgical theory, this study analyzes data from 8,499 completed campaigns on China’s Waterdrop Charity platform from 2018 to 2023. We conduct regression analyses of potential performance determinants to explore, across three dimensions, the key factors shaping medical crowdfunding performance. The results show that fundraising performance is positively associated with dynamic frontstage updates and with distinctive (non-formulaic) narratives; the experience of backstage charitable organizations amplifies the positive effect of dynamic updates; and donors’ sharing/reposting behavior serves as a transmission mediator. Building on these findings, we offer short- and long-term recommendations to optimize performance, improve the medical crowdfunding model, and promote the sustainable development of philanthropy.

## 1 Introduction

With global population aging and the rising incidence of chronic diseases, healthcare expenditures continue to climb (1), imposing a heavy burden on individuals, families, and social health insurance systems. Traditional charity, which relies on government leadership and institutional operations, faces limitations in coverage and responsiveness and thus struggles to meet increasingly diverse public needs (2). Against this backdrop, the spread of internet technologies has brought disruptive change to philanthropy, giving rise to medical crowdfunding as an innovative financing model. By filling gaps in medical payments and promoting the health and well-being of vulnerable groups, it aligns with the Sustainable Development Goals’ core aims of “good health and well-being” and “reduced inequalities.” Through online platforms, small contributions from the public are aggregated, breaking traditional constraints of time, space, and geography; philanthropy at one’s fingertips and participation by all have become a reality. This helps patients alleviate financial pressure and, in essence, represents a new form of social mutual aid in the digital era, becoming an important supplement to a multi-tiered medical security system (3). Thanks to its convenience and low barriers to entry, medical crowdfunding reduces promotional costs for fundraising projects, expands fundraising channels, and attracts broad participation from ordinary citizens, showing substantial potential and vitality in resource mobilization and developing rapidly across many countries. In the United States, the GoFundMe platform holds the largest global market share for medical crowdfunding and is relatively mature (4). In some developing countries such as India, where medical insurance systems are weak, medical crowdfunding has even become a substitute for insurance, underscoring the need for legal oversight and regulation (5). From a sustainable development perspective, the ideal state of medical crowdfunding is a long-term, stable, and self-sustaining ecosystem, one that cultivates public engagement under standardized platform governance and ensures efficient fundraising and equitable allocation of resources.

The growth of internet-based public welfare crowdfunding platforms has improved access to donation channels; however, technology-enabled medical crowdfunding still faces pronounced disparities in fundraising performance. As a middle-income country, China has seen a steady expansion of charitable giving, yet the policy environment remains immature and actual fundraising levels are uneven. Data from a major Chinese medical crowdfunding platform for 2017–2020 show that only 6% of projects reached their fundraising goals, with a standard deviation of amounts raised as high as 153,001.27, indicating generally low fundraising levels (6). A salient “Matthew effect” differentiates high-quality from ordinary projects, suggesting that project characteristics and related factors may be decisive for fundraising success. Meanwhile, the virtual fundraising context exacerbates a crisis of trust: donors have difficulty verifying the authenticity of recipients’ financial conditions and medical needs, necessitating more proactive information disclosure by charitable organizations (7). In the context of ongoing digital transformation and heightened transparency in the philanthropic sector, probing the core determinants of medical crowdfunding performance and their underlying mechanisms can not only improve the success rate of individual campaigns but also help build a sustainable online philanthropic ecosystem that complements and strengthens the social health insurance system over the long term.

This study makes several contributions for researchers, governments, crowdfunding platforms, and charitable organizations. First, it extends the application of dramaturgical theory by incorporating it into an analytical framework for medical crowdfunding. Second, it focuses on charitable organizations’ proactive interactive behaviors and on the distinctiveness of project content, while controlling for covariates commonly used in prior research; it also examines the moderating roles of organizational credibility and experience, yielding a more systematic analysis than earlier studies. Third, it highlights the mediating role of donor interaction, showing that donor sharing/reposting functions as a secondary diffusion mechanism that effectively drives performance gains. Finally, based on the empirical results, the study proposes targeted recommendations to optimize performance, offering clear practical implications.

The remainder of this paper proceeds as follows. Section 2 reviews the literature. Section 3 presents the theoretical framework and research hypotheses. Section 4 describes the research methods and empirical analyses, including sample selection, variable construction, and model specification. Section 5 reports the empirical results and robustness checks. Section 6 offers discussion and performance optimization recommendations. Section 7 outlines limitations and future directions. Section 8 concludes.

## 2 Literature review

The concept of “crowdfunding” was first introduced by the American scholar Michael Sullivan, referring to a form of financing oriented toward the public. The earliest documented case dates to 1884, when funds were raised for the construction of the Statue of Liberty, while internet-based crowdfunding in the modern sense began in 2009 and has since entered the public eye as a new financing model.

Regarding typologies of internet crowdfunding, the World Bank classifies crowdfunding into five systems: donation-based, reward/pre-sale–based, lending-based, equity-based, and royalty/ownership-based models. In terms of financing mechanism design, Cumming et al. (8) systematically elaborates two core mechanisms, Keep-It-All (KIA) and All-Or-Nothing (AON): under KIA, initiators may retain all funds raised regardless of whether the target is met; under AON, the preset goal must be fully achieved for the initiator to receive the funds. Studies show that reward-based projects are more likely to adopt AON, whereas public-welfare projects more often choose KIA (8). From a user-behavior perspective, Hemer (9) divides crowdfunding into five major modes, donation, sponsorship, pre-sale, lending, and equity. The medical crowdfunding examined in this study falls under the donation-based model (9).

As an emerging mechanism for medical finance, medical crowdfunding restructures traditional charitable giving through social media and online platforms. Its performance drivers and sustainability have likewise become focal points in the literature, with key determinants identified at the levels of project characteristics, initiator attributes, and social networks. At the project level, Wang et al. (10) finds using Kickstarter data that setting reasonable goals and timelines significantly increases success rates; the frequency of monetary-evidence disclosure on the project page is positively associated with completion rates (11); and moderately framed hardship narratives increase donation intention by 27% (12). Focusing on initiator attributes, Durand et al. (13) show in the U. S. Watsi case that the initiator’s social-network size and credibility endorsements directly affect fundraising efficiency (13). Social networks exert a pronounced amplifying effect: initial donations from strong ties account for 62% of total funds, while diffusion through weak ties expands the reach by a factor of 4.7 (14).

Currently, the sustainable development of medical crowdfunding faces twin challenges of wide performance disparities across projects and donor fatigue. Although prior studies have accumulated findings on performance drivers, most adopt narrow theoretical lenses, such as signaling theory or prosocial behavior, leading to limited cross-disciplinary integration and a lack of unified frameworks. Moreover, factor analyses are often fragmented, examining single variables (e.g., project features or initiator attributes) or simple linear relationships, without revealing synergistic effects among multiple drivers. While existing work has laid groundwork on model typologies and the identification of key elements in medical crowdfunding, the overall discussion remains dispersed and under-theorized with respect to underlying mechanisms. In response, this study introduces dramaturgical theory as an analytical framework, viewing medical crowdfunding as a form of “theatrical interaction,” with initiators as “actors” and donors as the “audience.” We integrate multi-dimensional variables including narrative distinctiveness (textual distinctiveness), rare-disease status, and initiator behaviors, to move beyond the limitations of single-factor linear analyses. We then construct a “frontstage performance–backstage support–audience interaction” framework to address gaps in theoretical explanation. By clarifying the internal logic of how medical crowdfunding performance is generated and by proposing systematic optimization, the study offers a fresh perspective on performance research and explores practical, high-impact strategies. These findings provide actionable recommendations for charitable organizations, platforms, and government, helping to steer medical crowdfunding toward greater equity, efficiency, and sustainability.

## 3 Theoretical framework and research hypotheses

### 3.1 Theoretical framework

Dramaturgical theory, proposed by sociologist Erving Goffman, analogizes social interaction to theatrical performance and emphasizes how individuals shape others’ impressions through “frontstage” and “backstage” behaviors (15). In The Presentation of Self in Everyday Life, Goffman argues that people engage in “impression management” via systems of symbols, such as language, gestures, and props, to meet social expectations or achieve specific goals. Individuals display norm-conforming conduct in public settings (the frontstage), while in private spaces (the backstage) they may reveal a more authentic self.

The theory comprises six core elements: performance (behavior presented before observers); team (a collaborative group that sustains the performance); region (the spatial or situational division between frontstage and backstage); discrepant roles (participants who may disrupt the performance); out-of-character communication (private interactions occurring outside formal roles); and techniques of impression management (e.g., idealization and mystification). Goffman underscores that social interaction is essentially a “collaborative performance,” maintained through tacit coordination, with audience feedback being pivotal to success.

Dramaturgical theory has been extended in communication studies, organizational behavior, and management. In the context of medical crowdfunding, it similarly provides a distinctive lens on initiator–donor interaction. A crowdfunding campaign is, in essence, a performance: the initiator crafts a carefully designed frontstage presentation to attract donor “box office,” while coordination between frontstage and backstage builds trust and helps achieve performance goals. The dramaturgical perspective also complements other theories. Trust theory highlights the importance of credibility and institutional safeguards in crowdfunding (16). Signaling theory addresses information asymmetries; in our research design, effective frontstage and backstage behaviors serve as signals that foster audience trust. Likewise, from a behavioral-economics perspective, narrative framing and emotional appeals influence donor decision-making (17), which we also examine. By integrating these interdisciplinary insights with a dramaturgical perspective and utilizing the metaphor of performance, we connect project impression management, the cultivation of trust in charitable organizations, and audience psychological engagement, enabling us not only to explain which factors influence crowdfunding performance but also to clarify why.

#### 3.1.1 Frontstage dynamic continuity: regular information updates

Within dramaturgical theory, a “dynamic, continuous” frontstage performance stresses sustained interaction between the actor and the audience to keep momentum. In crowdfunding, proactive behaviors such as posting updates are crucial for boosting engagement and improving the odds of success. Prior research consistently shows that timely updates significantly and positively influence both the number of investments/donations and total funds raised, especially when updates are clear, prompt, and oriented toward new developments or campaign progress rather than merely team or product details (18, 19). The impact of updates is greatest early in a campaign and tends to diminish as frequency increases or as the campaign advances (20). Strategically deploying updates to enhance team identity and confidence in future developments increases investment decisions (21). In medical crowdfunding, update content that appeals to credibility or compassion also promotes giving (22). In short, strategic, well-timed, and deliberate updates are key tools for reducing uncertainty, encouraging herd behavior, and maximizing outcomes. In this domain, the number of project updates is central to cultivating the image of an “active performer”: frequently sharing treatment progress and fund usage simulates a drama that “continues to advance the plot,” strengthening donors’ perceptions of authenticity. Accordingly, we hypothesize:

*H1*: Frontstage Dynamic continuous behavior is positively associated with fundraising performance.

#### 3.1.2 Frontstage static special plot: differentiated narrative

The most immediate content display of a crowdfunding project is its detail page. In dramaturgical terms, the static frontstage emphasizes the “uniqueness of the script,” which, in medical crowdfunding, calls for breaking textual homogeneity through distinctive narrative. Kahneman (23) shows that human attention is limited; in crowdfunding, textual distinctiveness materially affects outcomes, and detail descriptions should foreground creativity and uniqueness (24). Hirshleifer and Teoh (25) likewise demonstrate that information salience directly shapes attention on platforms. Differences in the composition and distinctiveness of project text are critical drivers of success: higher textual distinctiveness helps avoid formulaic “performances,” capture attention, and deepen memory. Both hard and soft information in the narrative can influence performance; negative-emotion cues can sometimes increase donations (26, 27). Given that medical crowdfunding centers on patients, particularly those with rare diseases, who typically face higher costs and greater therapeutic difficulty, the low-probability, high-adversity nature of rare conditions readily elicits sympathy and emotional resonance. A special plot is thus pivotal for generating affective impact, and more negative framing can, at times, yield more favorable results (28). The more distinctive the storyline, the more it can break conventional frames and stimulate giving. In this study, the static special plot dimension comprises two elements: (a) textual distinctiveness and (b) beneficiaries’ rare-disease status. We therefore propose:

*H2*: Frontstage Static special plot is positively associated with fundraising performance.

*H2a*: Higher textual distinctiveness is associated with higher fundraising performance.

*H2b*: Campaigns for beneficiaries with rare diseases achieve significantly higher fundraising performance than those without rare-disease status.

#### 3.1.3 Backstage support capabilities: credibility and narrative continuity

In dramaturgical theory, backstage support provides the experiential foundation for frontstage performance; the background and attributes of charitable organizations are thus vital to campaign success. Evidence indicates that founders’ cultural background, reputation, and social capital positively affect outcomes, especially in cultural, creative, and medical crowdfunding (29). Projects initiated by reputable organizations or charities often outperform those launched by individuals, primarily because potential backers perceive greater trustworthiness and credibility (30). Embedded relationships and strong social ties increase both the likelihood and amount of funding (31, 32). Moreover, initiators’ commitment and narrative ability are crucial for building trust and attracting support (33, 34). Overall, from organizational affiliations and social capital to personal presentation and storytelling, initiator attributes are key determinants of performance. In medical crowdfunding, alignment between the initiating and implementing charities reflects “cast coordination.” When these entities are misaligned, the backstage team must reconcile information, incurring communication costs and risking discontinuities in frontstage updates that may diverge from actual progress. When they are aligned, shorter information chains help ensure timely, accurate updates and bolster donors’ sense of authenticity. The initiator’s number of prior projects indicates a “track record,” i.e., institutional social capital. Prior findings suggest that repeat fundraisers with social capital are more successful than novices (35, 36). Charities with more prior projects resemble seasoned actors: their accumulated indirect experience can optimize current dynamic-performance strategies (37), calibrate update frequency, build audience trust, and amplify positive performance effects. Notably, our empirical focus is on dynamic continuity rather than static content: by the nature of staged performance, backstage capacity more directly enhances the continuity and credibility of frontstage actions, whereas static special plots are tied to beneficiary circumstances and, once posted, are not readily revised. In this study, backstage support capacity has two facets: (a) initiator–executor alignment and (b) the initiator’s number of prior projects. Thus, we hypothesize:

*H3*: Backstage support capacity positively moderates the relationship between frontstage dynamic continuous behavior and fundraising performance.

*H3a*: When the initiator and executor are aligned, the positive effect of frontstage dynamic continuous behavior on fundraising performance is significantly stronger than when they are not aligned.

*H3b*: The greater the initiator’s number of prior projects, the stronger the positive effect of frontstage dynamic continuous behavior on fundraising performance.

#### 3.1.4 Audience interaction: secondary dissemination of the performance

In dramaturgical terms, “audience interaction” is the key conduit for transferring performance value. Herding effects shape how donors interact with campaigns and decide whether to give (38). Donor interaction, such as reposting campaigns or sharing on social media, plays an important role in both campaign success and donor retention. Social-media activity, including the number of reposts and shares, expands visibility, attracts additional donors, and promotes campaign success (14, 39), underscoring the importance of social ties and communication (40). Donor retention and repeated giving are also linked to sustained engagement, including social connections and prior platform interactions (41). In sum, encouraging interaction (reposts, comments, and social sharing) can markedly increase the reach and effectiveness of crowdfunding. In medical crowdfunding, reposting signifies a shift from passive viewing to active participation, enabling secondary diffusion of the frontstage performance and operationalizing “emotional resonance.” Charitable organizations should harness the propagation effects of social networks to enhance fundraising efficiency (42). Analyses of charitable crowdfunding on Facebook show a positive association between the number of shares and donation revenue; more frequent sharing significantly increases the scale of donations (43). When donors, after viewing project details, choose to repost, they help the frontstage performance “break out of its initial audience bubble,” thereby influencing fundraising performance and forming a mediating path of “frontstage performance → audience interaction → fundraising performance.” In this study, the frontstage performance dimension comprises three elements: (a) number of project updates, (b) textual distinctiveness, and (c) rare disease in beneficiary patients. We therefore hypothesize:

*H4*: Audience interaction behavior mediates the relationship between frontstage performance and fundraising performance.

*H4a*: Audience interaction behavior mediates the relationship between number of project updates and fundraising performance.

*H4b*: Audience interaction behavior mediates the relationship between textual distinctiveness and fundraising performance.

*H4c*: Audience interaction behavior mediates the relationship between rare disease in beneficiary patients and fundraising performance.

**Figure 1:**
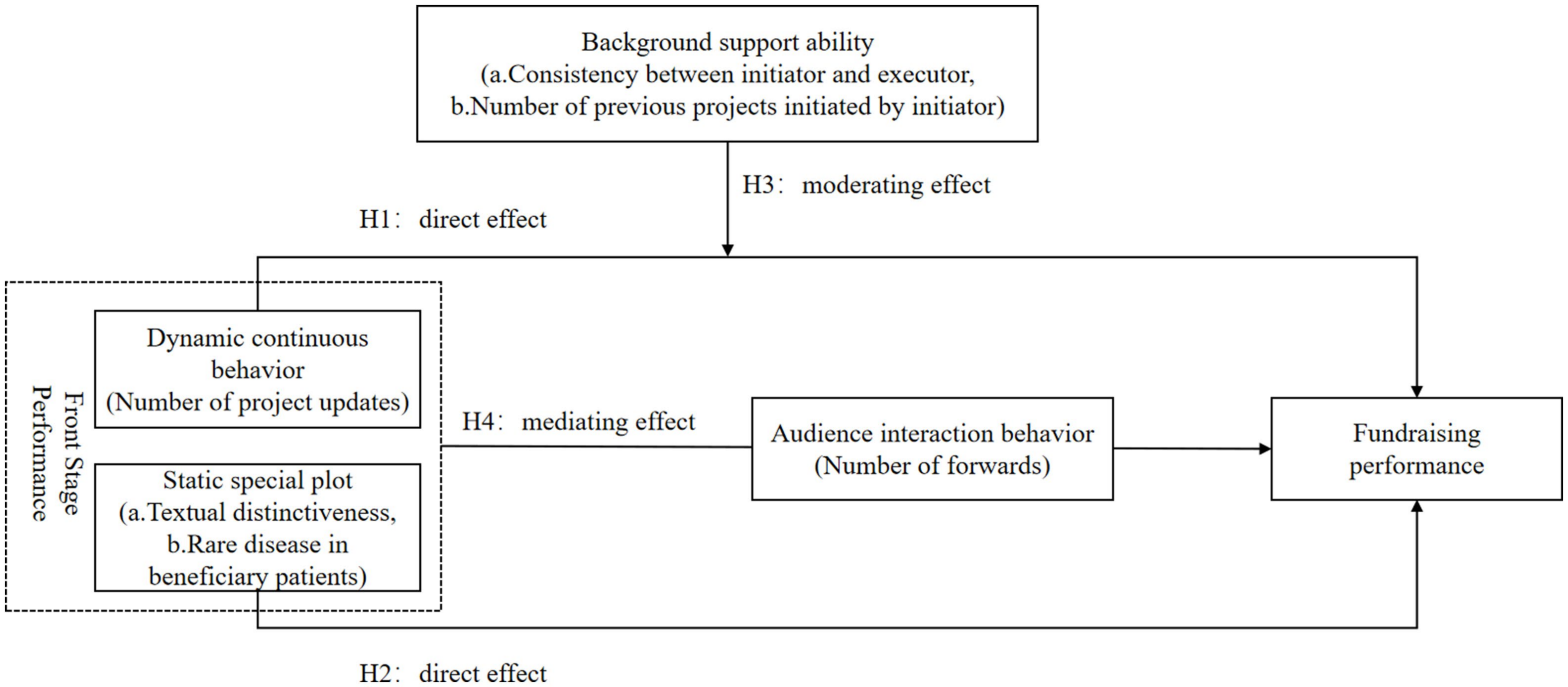
Theoretical analysis framework diagram.

Figure 1 presents the theoretical framework of this study: frontstage factors directly affect fundraising performance; backstage factors strengthen the effect of updates; and audience sharing behavior transmits and amplifies the impact of frontstage performance to achieve broader outcomes.

## 4 Methodology

### 4.1 Data collection

As of the end of August 2024, China had 15,334 registered charitable organizations, of which 3,250 possessed public fundraising credentials. To accommodate the deepening application of internet technologies in the charitable sector, China has successively promulgated the Charity Law of the People’s Republic of China and the Administrative Measures for Public Fundraising Platform Services since 2016. At present, 29 internet-based public fundraising information platforms provide professional services for charities and are incorporated into statutory regulatory frameworks, effectively advancing the standardization of China’s online fundraising environment.

This study draws data from Waterdrop Charity (Shuidi Gongyi), one of China’s leading internet public-welfare platforms that provides convenient fundraising services for individuals and nonprofit organizations. As of June 30, 2025, the platform had published over 15,500 public-welfare projects, attracted more than 73.16 million donors, and raised in excess of CNY 1.374 billion, indicating broad public participation and influence. Waterdrop Charity features a high level of transparency and standardization: all projects must disclose structured information at launch, such as a project overview, budget, and implementing agency, ensuring that potential donors receive consistent and transparent project information.

We employ a cross-sectional research design with the individual medical crowdfunding project as the unit of analysis. The dataset consists of publicly available secondary data on projects initiated and completed on Waterdrop Charity between 2018 and 2023. We record only each project’s final outcome and do not track within-project changes across years. Using a Python-based automated web crawler, we collected an initial sample of 9,189 projects. During data cleaning, we applied stringent screening to remove test projects that did not actually conduct fundraising and to exclude cases with missing key information, specifically, those with zero funds raised, zero recorded fundraising duration, or empty project descriptions. The final analytic sample comprises 8,499 valid observations, providing a reliable basis for the subsequent empirical analyses.

### 4.2 Variable construction

#### 4.2.1 Dependent variable

The dependent variable in this study is comprehensive fundraising performance for medical crowdfunding campaigns (FP). Existing studies adopt various indicators to measure performance, including the actual amount raised, fundraising success rate, percentage of goal achieved, and number of donors. For example, Calic and Shevchenko (44) uses the number of donors, total funds raised, and goal attainment as separate dependent variables. Li et al. (45) standardize five indicators to construct a composite performance index for multivariate analysis. To capture the multidimensional nature of performance, we adopt a principal component analysis (PCA) approach using three standardized indicators: the amount raised, number of donations, and fundraising percentage. *Z*-score standardization is applied to eliminate scale differences. As shown in Table 1, the KMO value is 0.7, indicating that the data are suitable for PCA. The Bartlett’s test for sphericity is significant (*p* < 0.001), further confirming appropriateness for factor extraction.

**Table 1:** KMO and Bartlett’s test of sphericity.

KMO value		0.7
Bartlett’s test of sphericity	Approximate chi-square	30536.298
	df	3
	*P*	0.000

Based on the eigenvalues and factor loadings, a single principal component was extracted with a cumulative explained variance of 89.6%. The respective loadings for the three indicators are 0.594, 0.588, and 0.548. This composite index effectively captures the overall performance of each campaign by integrating the core dimensions of crowdfunding success.

#### 4.2.2 Independent variables

Our explanatory variables capture the frontstage performance dimension, namely dynamic continuous behavior and the static special plot.

For dynamic continuous behavior, we measure the number of project updates posted before the campaign concluded (NPU) (31). Following Mollick (31) empirical work on Kickstarter, this variable is taken directly from the platform: a higher NPU indicates more frequent interaction between the charitable organization and its audience during the fundraising period.

For the static special plot, we focus on two aspects. First, textual distinctiveness (TD) refers to the degree to which the project description contains multi-dimensional information, such as details on the patient’s condition and family hardship, and is differentiated from similar content. In an environment saturated with information, a distinctive project narrative is more likely to capture the limited attention of potential donors (25). To quantify this, we measured how different a project’s descriptive text was from the average text on the platform. Our approach builds on established methods in computational linguistics for assessing document uniqueness (46, 47). Operationally, we removed English letters, digits, and symbols, and constructed a composite Chinese stopword list by integrating the HIT stopword list, the “Chinese Stopword List,” the Baidu stopword list, and the Sichuan University Machine Intelligence Laboratory stopword list. Then, using the sklearn package, we converted each description into a TF-IDF vector, which represents the text numerically based on word importance. Finally, we calculated the cosine similarity of each project’s vector against the average of all other project vectors. The textual distinctiveness (TD) was defined as 1 minus this average similarity score. A higher TD value thus indicates a more unique and less formulaic narrative. Second, we code rare disease in beneficiary patients (RD) as another static dimension. Following Ba et al. (30), we identify RD based on text extraction from the project-details field, assigning 1 if the beneficiary is described as having a rare disease and 0 otherwise (30, 48).

#### 4.2.3 Moderator and mediator variables

The moderator variables capture the backstage support capacity. Guided by our hypotheses and data availability, we focus on two measures: consistency between initiator and executor (IE) and number of previous projects initiated by initiator (NPP). Building on Vincenzo’s finding that repeat fundraisers can leverage supporters’ social ties (35), we code IE = 1 if the charitable organization that initiates the crowdfunding campaign is also the implementing agency; otherwise, IE = 0. Number of previous projects initiated by initiator (NPP) operationalizes organizational experience, computed from the timeline of past campaigns. A higher NPP indicates a more experienced fundraiser.

The mediating variable represents the audience-interaction dimension. On the Waterdrop Charity platform, visitors can forward campaign links to external channels. We recorded, at each campaign’s end, the number of times it was forwarded to outside platforms. This number of forwards (NF) is the cumulative number of shares and reflects the extent of secondary diffusion by the audience.

#### 4.2.4 Control variables

Consistent with dramaturgical theory, the stage setting and props (the basic scene and cues surrounding a performance) can influence the eventual box office. Following prior research (49), we therefore control for readily observable project-page information to isolate the effects of our focal variables: fundraising time (FT), target amount (lnTA), the number of pictures (NP), length of details (lnLD), and length of introduction (LI).

Definitions and coding of all variables are summarized in Table 2.

**Table 2:** Description of measurements of variables collected in this study.

Variable type	Theater segment of dramaturgical theory	Core logic	Variable name	Variable declaration	Source
Explained variable	Final ticket office	Measurement of performance results	Fundraising performance (FP)	A composite indicator of the amount raised, the percentage of fundraising and the number of donations	Adapted from Multi-Indicator Performance Measurement (44, 45)
Explanatory variables	Performing by front-stage actors	Dynamic continuous behavior	Number of project updates (NPU)	Number of progress updates before the end of the project	Platform data, frequently updated as a participation signal (18)
		Static special plot	Textual distinctiveness (TD)	The information content that distinguishes the project text from that of other projects	Referring to the practices of Tata and Patel (46) and Kim et al. (47)
			Rare disease in beneficiary patients (RD)	Beneficiaries suffering from rare diseases 1, the rest 0	Through keyword recognition (30, 48)
Moderator variables	Background support ability	Credibility and coherence of dynamic continuous behavior	Consistency between initiator and executor (IE)	Institutions consistent note 1, the rest note 0	Platform Data (30)
			Number of previous projects initiated by initiator (NPP)	Number of projects initiated prior to the launch of the project	Based on the sample time calculation, refer to the study by Butticè et al. (35)
Mediator variable	Audience interaction behavior	Audience’s second diffusion of performance	Number of forwards (NF)	Number of times forwarded before the end of the project	Platform data, representing dissemination data (14, 39)
Control variables	Stage setting	The basic scene and props of the performance	Fundraising time (FT)	Duration of project fund-raising	Platform data, control time span
			Target amount (lnTA)	The target amount of the project raised, take the natural logarithm	Platform data, controlling project scale (49)
			Number of pictures (NP)	Number of images in project details	Platform data and visual information can affect attractiveness (28, 37)
			Length of details (lnLD)	The number of characters in the project details, take the natural logarithm	Platform data, control of the amount of information provided (49)
			Length of introduction (LI)	The number of characters in the project title	Platform data, control the conciseness and detail of the title (49)
			Fixed year (year)	Year of project initiation 2018–2023	Control the time trend
			Fixed province (Pov)	According to China’s administrative divisions 11–65	Control area factors

### 4.3 Model specification

This study employs multiple regression analysis to examine interaction effects among key variables. Based on the proposed hypotheses, the empirical models are constructed as follows:

Model 1 (Equation 1) introduces explanatory and control variables to test the main effects (Hypotheses H1 and H2). The dependent variable is fundraising performance (FP), with *α*₀ as the constant term. Independent variables include the number of project updates (NPU), textual distinctiveness (TD), and rare disease in beneficiary patients (RD). Control variables include fundraising duration (FT), log-transformed target amount (lnTA), number of images (NP), log-transformed text length (lnLD), introduction length (LI), project launch year (Year), and beneficiary’s province (Pov). The model is specified as follows:


$$\begin{array}{l} \mathrm{FP}=\alpha_{0}+\alpha_{1}\mathrm{NPU}+\alpha_{2}\mathrm{TD}+\alpha_{3}\mathrm{RD}+\alpha_{4}\text{FT}\\ +\alpha_{5}\text{lnTA}+\alpha_{6}\mathrm{NP}+\alpha_{7}\text{lnLD}+\alpha_{8}\mathrm{LI}+\alpha_{9}\text{Year}+\alpha_{10}\mathrm{Pov}+\varepsilon_{i} \end{array}$$ (1)

To test Hypothesis H3 on the moderating role of backstage support, Model 2 (Equation 2) adds the variable of consistency between initiator and executor (IE) and its interaction with NPU (mod1), while Model 3 (Equation 3) includes number of previous projects initiated by initiator (NPP) and its interaction with NPU (mod2):


$$\begin{array}{l} \mathrm{FP}=\alpha_{0}+\alpha_{1}\mathrm{NPU}+\alpha_{2}\mathrm{IE}+\alpha_{3}\mathrm{mod}1+\alpha_{4}\mathrm{TD}\\ +\alpha_{5}\mathrm{RD}+\alpha_{6}\text{FT}+\alpha_{7}\text{lnTA}+\alpha_{8}\mathrm{NP}+\alpha_{9}\text{lnLD}\\ +\alpha_{10}\mathrm{LI}+\alpha_{11}\text{Year}+\alpha_{12}\mathrm{Pov}+\varepsilon_{i} \end{array}$$ (2)

$$\begin{array}{l} \mathrm{FP}=\alpha_{0}+\alpha_{1}\mathrm{NPU}+\alpha_{2}\mathrm{NPP}+\alpha_{3}\mathrm{mod}2+\alpha_{4}\mathrm{TD}\\ +\alpha_{5}\mathrm{RD}+\alpha_{6}\text{FT}+\alpha_{7}\text{lnTA}+\alpha_{8}\mathrm{NP}+\alpha_{9}\text{lnLD}\\ +\alpha_{10}\mathrm{LI}+\alpha_{11}\text{Year}+\alpha_{12}\mathrm{Pov}+\varepsilon_{i} \end{array}$$ (3)

To test Hypothesis H4 the mediating role of audience interaction, we employed the Bootstrap method provided by Hayes (50) using the SPSS macro Process. Model (4) (Equation 4) represents the effect equation of X on M, while Model (5) (Equation 5) represents the effect equation of X on Y after controlling for M. In these models, NF denotes number of forwards:


$$\begin{array}{l} \mathrm{NF}=\alpha_{0}+\alpha_{1}\mathrm{NPU}+\alpha_{2}\mathrm{TD}+\alpha_{3}\mathrm{RD}+\alpha_{4}\text{FT}\\ +\alpha_{5}\text{lnTA}+\alpha_{6}\mathrm{NP}+\alpha_{7}\text{lnLD}+\alpha_{8}\mathrm{LI}+\alpha_{9}\text{Year}+\alpha_{10}\mathrm{Pov}+\varepsilon_{i} \end{array}$$ (4)

$$\begin{array}{l} \mathrm{FP}=\alpha_{0}+\alpha_{1}\mathrm{NF}+\alpha_{2}\mathrm{NPU}+\alpha_{3}\mathrm{TD}+\alpha_{4}\mathrm{RD}+\alpha_{5}\text{FT}\\ +\alpha_{6}\text{lnTA}+\alpha_{7}\mathrm{NP}+\alpha_{8}\text{lnLD}+\alpha_{9}\mathrm{LI}+\alpha_{10}\text{Year}+\alpha_{11}\mathrm{Pov}+\varepsilon_{i} \end{array}$$ (5)

## 5 Empirical results and analysis

This study, grounded in dramaturgical theory, employs a multivariate regression model to examine the determinants of medical crowdfunding performance. It focuses on the influence of initiators’ dynamic updates, project content distinctiveness, the moderating role of organizational experience, and the mediating role of donor interaction.

### 5.1 Descriptive statistics and correlation analysis

Data analysis was conducted using STATA. Table 3 reports the descriptive statistics of key variables. The fundraising performance (FP) variable exhibits a skewed distribution, as indicated by its median and standard deviation, suggesting substantial variation in fundraising outcomes across projects. The number of project updates (NPU) has a mean of 4.318 and a median of 3. While some projects had no updates, others were updated up to 54 times, reflecting varied levels of engagement. Frequent updates may signal proactive project management and active donor communication by charitable organizations. Textual distinctiveness (TD) shows a relatively concentrated distribution but includes several extreme values. The rare disease indicator (RD), a defining feature in medical crowdfunding, reveals that only 6.3% of beneficiaries are diagnosed with rare diseases, highlighting this subgroup’s analytical significance due to its exceptional characteristics.

**Table 3:** Results of descriptive statistics.

Variable	*N*	Mean	p25	p50	p75	SD	Min	Max
Fundraising performance (FP)	8,499	0	−0.864	−0.693	0.0130	1.640	−0.905	15.01
Number of project updates (NPU)	8,499	4.318	2	3	6	3.939	0	54
Textual distinctiveness (TD)	8,499	0.500	0.381	0.518	0.627	0.164	0	1
Rare disease in beneficiary patients (RD)	8,499	0.0630	0	0	0	0.242	0	1
Fundraising time (FT)	8,499	263.2	151	295	365	135.2	17	1,621
Target amount (lnTA)	8,499	12.56	12.21	12.61	12.90	0.585	9.259	15.43
Number of pictures (NP)	8,499	7.267	5	7	9	3.343	0	31
Length of details (lnLD)	8,499	6.988	6.671	7.005	7.324	0.478	2.565	8.652
Length of introduction (LI)	8,499	20.73	18	22	25	4.708	0	27
Year	8,499	2020	2020	2020	2021	0.968	2018	2023
Pov	8,499	1.742	1	1	2	0.968	1	4

Table 4 presents the Pearson correlation coefficients and significance levels among key variables. All independent variables are positively correlated with fundraising performance, offering preliminary support for Hypotheses H1 and H2. The table also includes results from multicollinearity diagnostics. The average Variance Inflation Factor (VIF) is 1.33, with all VIF values well below the threshold of 10, indicating no serious multicollinearity concerns and confirming the dataset’s suitability for regression analysis.

**Table 4:** Correlation and multicollinearity tests.

Variable	FP	NPU	TD	RD	FT	LnTA	NP	lnLD	LI	VIF
Fundraising performance (FP)	1									
Number of project updates (NPU)	0.438***	1								1.020
Textual distinctiveness (TD)	0.036***	−0.051***	1							1.030
Rare disease in beneficiary patients (RD)	0.130***	0.069***	0.023**	1						1.030
Fundraising time (FT)	0.069***	0.109***	0.100***	0.003	1					1.140
Target amount (lnTA)	0.196***	0.030***	0.023**	0.091***	0.291***	1				1.180
Number of pictures (NP)	0.042***	0.032***	−0.037***	0.067***	0.211***	0.258***	1			2.050
Length of details (lnLD)	0.066***	0.046***	0.036***	0.125***	0.188***	0.290***	0.707***	1		2.110
Length of introduction (LI)	0.023**	0.045***	−0.004	0.050***	0.066***	0.111***	0.186***	0.219***	1	1.060

Standard errors in parentheses.

*p < 0.1, **p < 0.05, ***p < 0.01.

### 5.2 Main regression results

The main effects regression was conducted to examine the direct influence of key variables on medical crowdfunding performance. Model (1) in Table 5 presents the baseline results.

**Table 5:** Main regression results (hypotheses H1 and H2).

Variable	Model (1)
	Fundraising performance (FP)
Number of project updates (NPU)	0.204***
	(0.004)
Textual distinctiveness (TD)	0.329***
	(0.083)
Rare disease in beneficiary patients (RD)	0.531***
	(0.064)
Fundraising time (FT)	−0.001***
	(0.000)
Target amount (lnTA)	0.410***
	(0.030)
Number of pictures (NP)	0.006
	(0.007)
Length of details (lnLD)	0.043
	(0.048)
Length of introduction (LI)	−0.003
	(0.003)
Year	Yes
Pov	Yes
_cons	−5.974***
	(0.448)
*N*	8,499
r2	0.286
r2_a	0.282

Standard errors in parentheses.

***p < 0.01.

The coefficient on the number of project updates (NPU) is 0.204 and statistically significant at the 1% level, indicating that, holding other factors constant, each additional update is associated with an increase of about 0.204 units in fundraising performance. This sizable effect highlights the positive value of frequent updates: the more often campaigns are updated, the more public attention and donations they attract, thereby markedly improving fundraising performance, which supports H1. As campaign initiators and the parties directly responsible to patients, charitable organizations should supplement the initial project information with ongoing updates on treatment progress and patient status so that donors continuously perceive interaction value.

The coefficient on textual distinctiveness (TD) is 0.329 and significant at the 1% level. Controlling for other variables, a one–standard-deviation increase in TD is associated with an expected 0.329-unit rise in fundraising performance, indicating that narratives rich in distinctive information help attract donations; H2a is thus supported. In addition, the coefficient on rare disease in beneficiary patients (RD) is 0.531 and significant at the 1% level, showing that campaigns involving beneficiaries with rare diseases score, on average, 0.531 units higher in fundraising performance than those without rare-disease status; H2b is therefore supported. Taken together, these findings validate H2 and suggest that, when publishing project details, charities should mine each patient’s distinctive circumstances and clearly flag rare-disease and other special conditions.

### 5.3 Moderating effect test

Table 6 reports the moderation tests. In Model (2), we introduce consistency between initiator and executor (IE) and the interaction term IE × NPU (mod1). The coefficient on mod1 is 0.050 and significant at the 1% level, indicating that IE positively moderates the relationship between project updates (NPU) and fundraising performance (FP). In other words, when a single charitable organization both initiates and implements the campaign, dynamic updates translate more effectively into donations. Donors are likely to view such information as more credible and coherent, and the trust and efficiency gains from organizational alignment make each update more potent in driving contributions. Accordingly, H3a is supported.

**Table 6:** Moderating effect regression results (Hypothesis H3).

Variable	Model (2)	Model (3)
	Fundraising performance (FP)	Fundraising performance (FP)
Number of project updates (NPU)	0.176***	0.193***
	(0.007)	(0.006)
Consistency between initiator and executor (IE)	0.003	
	(0.050)	
mod1 (IE × NPU)	0.050***	
	(0.008)	
Number of previous projects initiated by initiator (NPP)		−0.108
		(0.075)
mod2 (NPP × NPU)		0.035***
		(0.013)
Textual distinctiveness (TD)	0.338***	0.328***
	(0.083)	(0.083)
Rare disease in beneficiary patients (RD)	0.520***	0.535***
	(0.063)	(0.064)
Fundraising time (FT)	−0.001***	−0.001***
	(0.000)	(0.000)
Target amount (lnTA)	0.416***	0.411***
	(0.029)	(0.030)
Number of pictures (NP)	−0.001	0.006
	(0.007)	(0.007)
Length of details (lnLD)	0.080*	0.043
	(0.048)	(0.048)
Length of introduction (LI)	−0.002	−0.003
	(0.003)	(0.003)
Year	Yes	Yes
Pov	Yes	Yes
_cons	−6.261***	−5.955***
	(0.448)	(0.448)
*N*	849	8,499
r2	0.293	0.286
r2_a	0.289	0.282

Standard errors in parentheses.

***p < 0.01.

Similarly, Model (3) adds number of previous projects initiated by initiator (NPP) and the interaction NPP × NPU (mod2). The coefficient on mod2 is 0.035 and significant at the 1% level, showing that organizational experience strengthens the positive association between dynamic updates and FP. With accumulated experience, charities adjust their fundraising strategies more adeptly and tend to deploy updates more frequently and effectively. Thus, H3b is supported.

Taken together, these results confirm H3 on both fronts: backstage support capacity enhances the effectiveness of frontstage dynamic continuity.

### 5.4 Mediation effect test

Tables 7–9 present the results of the mediating effect test of audience interaction. Table 7 shows the results of testing the path from X to M in Model 4. The coefficients for NPU, TD, and RD are 0.513, 5.576, and 2.389, respectively, and are significant at the 1% level. Therefore, NPU, TD, and RD significantly influence the mediating variable NF. Table 8 shows the results for the path from M to Y in Model 5. The regression coefficient for NF is 0.051, significant at the 1% level. Thus, the mediating variable NF significantly influences the dependent variable FP. The output in Table 9 confirms the mediating effect, as the 95% confidence interval calculated via bootstrap does not include zero, indicating a significant mediating effect. Additionally, by comparing the total effect of X on Y without controlling for M, we find that after controlling for NF, the direct effects of NPU, TD, and RD on FP account for 85.635, 38.044, and 78.407%, respectively. The mediation effects of NF account for 14.365, 61.956, and 21.593%, respectively. This indicates that while NPU, TD, and RD directly influence FP, a portion of their effects is transmitted through NF, which partially mediates the effects of NPU, TD, and RD. Hypotheses H4a, H4b, and H4c are tested, and Hypothesis H4 holds. The mediating effect of number of forwards (NF) indicates that number of project updates, textual distinctiveness, and rare disease in beneficiary patients all directly enhance fundraising performance. Besides, through the mediating role of users forwarding projects, the impact is further amplified, helping break platform boundaries and significantly boost project performance.

**Table 7:** Mediation effect X-M path regression results (Hypothesis H4).

Model (4)	Coefficient	*s*	*t*	*p*	LLCI	ULCI
Number of project updates (NPU)	0.513	0.037	13.773	0.000	0.440	0.586
Textual distinctiveness (TD)	5.576	0.750	7.437	0.000	4.107	7.046
Rare disease in beneficiary patients (RD)	2.389	0.606	3.943	0.000	1.202	3.577
Fundraising time (FT)	−0.001	0.001	−1.231	0.219	−0.004	0.001
Target amount (lnTA)	4.327	0.269	16.099	0.000	3.800	4.854
Number of pictures (NP)	0.018	0.062	0.283	0.777	−0.104	0.139
Length of details (lnLD)	−0.345	0.440	−0.784	0.433	−1.208	0.518
Length of introduction (LI)	0.007	0.032	0.227	0.820	−0.055	0.069
Constant	−53.064	3.943	−13.456	0.000	−60.794	−45.333

LLCI and ULCI are abbreviations for the lower limit bootstrap confidence interval and upper limit bootstrap confidence interval, respectively.

**Table 8:** Mediation effect M-Y path regression results (Hypothesis H4).

Model (5)	Coefficient	*s*	*t*	*p*	LLCI	ULCI
Number of forwards (NF)	0.051	0.001	50.022	0.000	0.049	0.053
Number of project updates (NPU)	0.155	0.004	43.643	0.000	0.148	0.162
Textual distinctiveness (TD)	0.175	0.071	2.464	0.014	0.036	0.314
Rare disease in beneficiary patients (RD)	0.443	0.057	7.745	0.000	0.331	0.555
Fundraising time (FT)	0.000	0.000	−3.332	0.001	−0.001	0.000
Target amount (lnTA)	0.311	0.026	12.110	0.000	0.261	0.362
Number of pictures (NP)	−0.007	0.059	−1.128	0.260	−0.018	0.005
Length of details (lnLD)	0.007	0.042	0.173	0.862	−0.074	0.089
Length of introduction (LI)	−0.006	0.003	−2.060	0.039	−0.012	0.000
Constant	−4.711	0.376	−12.546	0.000	−5.448	−3.975

LLCI and ULCI are abbreviations for the lower limit bootstrap confidence interval and upper limit bootstrap confidence interval, respectively.

**Table 9:** Bootstrap test results for mediating effects (Hypothesis H4).

Independent variable	Path	Effect	se	*P*	LLCI	ULCI	Effect size
Number of project updates (NPU)	Overall effect	0.181	0.004	0.000	0.173	0.189	
	Direct effect	0.155	0.004	0.000	0.148	0.162	85.635%
	Mediation effect	0.026	0.010		0.015	0.051	14.365%
Textual distinctiveness (TD)	Overall effect	0.460	0.080	0.000	0.302	0.618	
	Direct effect	0.175	0.071	0.014	0.036	0.314	38.044%
	Mediation effect	0.285	0.106		0.148	0.527	61.956%
Rare disease in beneficiary patients (RD)	Overall effect	0.565	0.065	0.000	0.438	0.692	
	Direct effect	0.443	0.057	0.000	0.331	0.555	78.407%
	Mediation effect	0.122	0.064		0.048	0.292	21.593%

LLCI and ULCI are abbreviations for the lower limit bootstrap confidence interval and upper limit bootstrap confidence interval, respectively.

### 5.5 Robustness test

#### 5.5.1 Sample period adjustment

To verify the reliability of the empirical results, this study first conducts robust-ness testing by adjusting the sample period. Since 2020 marks the onset of the COVID-19 pandemic in China, a significant public health shock with widespread socio-economic impact, all data from 2020 were excluded. The regression results based on this adjusted sample are reported in Model (6) of Table 10. The signs and significance levels of all coefficients remain consistent with those in Model (1), all significant at the 1% level. This indicates that the core findings are robust and not fundamentally affected by the pandemic, confirming the stability of the main conclusions.

**Table 10:** Main regression results and regression results without samples from 2020.

Variable	Model (1)	Model (6)
	Fundraising performance (FP)	Fundraising performance (FP)
Number of project updates (NPU)	0.204***	0.187***
	(0.004)	(0.005)
Textual distinctiveness (TD)	0.329***	0.225**
	(0.083)	(0.098)
Rare disease in beneficiary patients (RD)	0.531***	0.394***
	(0.064)	(0.071)
Fundraising time (FT)	−0.001***	−0.000
	(0.000)	(0.000)
Target amount (lnTA)	0.410***	0.320***
	(0.030)	(0.031)
Number of pictures (NP)	0.006	0.004
	(0.007)	(0.007)
Length of details (lnLD)	0.043	0.081
	(0.048)	(0.051)
Length of introduction (LI)	−0.003	−0.006*
	(0.003)	(0.004)
Year	Yes	Yes
Pov	Yes	Yes
_cons	−5.974***	−5.099***
	(0.448)	(0.470)
*N*	8,499	5,158
r2	0.286	0.274
r2_a	0.282	0.268

Standard errors in parentheses.

***p < 0.01.

#### 5.5.2 Placebo test

To rule out potential confounding factors, a placebo test following Bertrand et al. (51) was performed. The three core independent variables were randomly reassigned values while keeping other control variables unchanged. These randomized variables were then regressed against fundraising performance. Table 11 shows three rounds of placebo regressions, with coefficients of −0.034, −0.033, and −0.034, respectively, all statistically insignificant. This suggests that the positive effects of the core variables on fundraising performance are unlikely due to sample selection bias.

**Table 11:** Placebo test regression results.

Variable	Model (7)	Model (8)	Model (9)
	Fundraising performance (FP)	Fundraising performance (FP)	Fundraising performance (FP)
placebo_NPU	−0.034		
	(0.029)		
placebo_TD		−0.033	
		(0.025)	
placebo_RD			−0.034
			(0.025)
Textual distinctiveness (TD)	0.357***		0.328***
	(0.091)		(0.080)
Rare disease in beneficiary patients (RD)	0.699***	0.507***	
	(0.069)	(0.061)	
Fundraising time (FT)	0.000	−0.001***	−0.001***
	(0.000)	(0.000)	(0.000)
Target amount (lnTA)	0.474***	0.401***	0.421***
	(0.033)	(0.029)	(0.029)
Number of pictures (NP)	0.009	0.004	0.006
	(0.008)	(0.007)	(0.007)
Length of introduction (LI)	0.002	−0.004	−0.003
	(0.004)	(0.003)	(0.003)
Length of details (lnLD)	−0.027	0.066	0.077*
	(0.053)	(0.046)	(0.047)
Number of project updates (NPU)		0.214***	0.216***
		(0.004)	(0.004)
Pov	Yes	Yes	Yes
Year	Yes	Yes	Yes
_cons	−6.242***	−5.762***	−6.255***
	(0.497)	(0.440)	(0.439)
*N*	8,499	8,499	8,499
r2	0.090	0.287	0.283
r2_a	0.085	0.283	0.279

Standard errors in parentheses.

*p < 0.1, ***p < 0.01.

**Figure 2:**
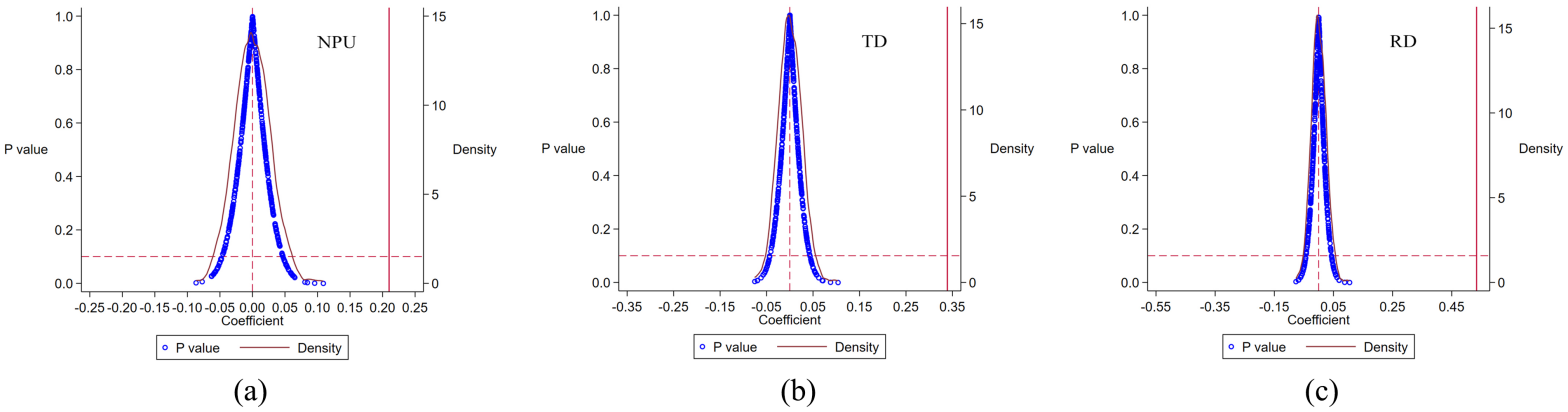
Placebo test distribution of NPU **(a)**, TD **(b)**, and RD **(c)** variables.

Additionally, 500 repeated random samplings were conducted to generate 500 estimates for each core variable. Kernel density plots for NPU, TD, and RD coefficients are presented in Figures 2a–c respectively. The distributions of the randomized coefficients are centered near zero, range between −0.1 and 0.1, and approximate normal distributions that do not cover the actual estimated values. These findings further confirm the robustness of the main regression results.

## 6 Discussion

### 6.1 Theoretical value

This study, grounded in dramaturgical theory, constructs a multiple regression model to empirically explore the factors influencing medical crowdfunding performance. It demonstrates the value of applying dramaturgical theory as a lens for understanding crowdfunding dynamics.

First, by viewing crowdfunding as a performance with frontstage and backstage components, we offer a holistic perspective that links previously isolated factors. While prior research has identified the importance of project updates (18, 19), narrative content (22), organizational credibility (30), and social sharing, these elements are typically studied in isolation. Using the dramaturgical framework, we show how they are interconnected: experienced, trusted organizations as backstage supporters enhance the effectiveness of frontstage dynamic behaviors such as project updates, and these updates, alongside the static narrative content, significantly impact the success of the performance.

Second, we observe that donor sharing acts as a form of social proof, mediating the relationship between the core variables and fundraising performance. The dramaturgical framework captures this as part of the performance, which means audiences are not passive observers but active participants who amplify the performance through sharing, validating it. This theoretical extension suggests that Goffman’s framework applies in the digital context, where online audiences can resonate with and amplify performances in unprecedented ways.

Furthermore, this integrated perspective helps explain the mechanisms behind fundraising success and enriches the application of signaling theory in crowdfunding contexts (16). Project updates and organizational consistency serve as signals of transparency and reliability. Our study quantifies and verifies the positive impact of updates on donor contributions and sharing behaviors, with the consistency and prior experience of the initiator and executor amplifying the effectiveness of these updates. This suggests that donors are particularly sensitive to trustworthiness. Additionally, our research aligns with and extends behavioral economics in charitable giving (17). Storytelling and emotional engagement significantly influence donor decisions. We confirm that unique, emotionally resonant narratives lead to higher donations.

### 6.2 Practical implications

Our findings offer practical recommendations for stakeholders in the internet-based medical crowdfunding ecosystem. These implications can be viewed from both short-term and long-term perspectives.

In the short term, the strategy should focus more on the project itself. As the initiator of the project, the charity organization needs to optimize its front-end performance: First, establish a regular update mechanism. For instance, charitable organizations can adopt a “Weekly Progress Record” template, including medical conditions and brief personal information about the patient’s family. When the project reaches significant milestones, such as when the patient completes surgery, the organization should promptly release detailed outcome reports documenting the surgical procedure’s completion. This transforms updates from mere tasks into program performance, ensuring donors remain engaged. Second, craft a distinctive narrative. Project descriptions should avoid generic templates. Tailor content based on each patient’s condition, highlighting specific, heart-wrenching details, such as the patient’s struggle to bear the family’s financial burden and their vision for a better future. Simultaneously, appropriately emphasize the rarity of the disease to enhance project recognition, using emotional conflict to deepen resonance. Crowdfunding platforms can empower short-term actions through design optimization, trigger automatic reminders for projects that have not been updated for a long time, use algorithms to identify rare diseases or unique narrative projects and increase their exposure, and set up diverse interactive functions such as likes and shares to facilitate user communication and lower the threshold for dissemination.

The long-term strategy starts from the environment. As a key force in macro-control and regulatory guidance, the government should strive to build a healthy, orderly and fair charitable environment to promote the sustainable development of medical crowdfunding, and regulate the behavior of public fundraising platforms and organizations in the field of public welfare crowdfunding from the top-level design level. On the one hand, formulate mandatory information disclosure policies, requiring projects to report progress and fund usage on a regular basis, and institutionalize dynamic transparency. On the other hand, a sound project supervision mechanism should be established, requiring the platform to supervise, review and publicize the fundraising progress, fund usage and other situations of the project. For projects that violate regulations, penalties should be imposed to safeguard the rights and interests of donors, ensure that crowdfunding projects operate on a standardized track, and promote the healthy and sustainable development of the charity sector. Crowdfunding platforms should provide personalized project recommendations based on user behavior data and collaborate with social media and communication applications to achieve cross-platform integration, simplifying the sharing and donation processes. Charitable organizations need to enhance their own capabilities, start from small-scale projects or cooperative activities to accumulate experience, gradually establish a good organizational structure and mature management model, and run through the entire process of medical crowdfunding from beginning to end, injecting strong impetus into the sustainable development of public welfare undertakings.

## 7 Limitations and future research

While this study provides a fresh perspective on medical crowdfunding, it has certain limitations. These limitations offer avenues for future research to solidify and address gaps in our work.

First, our data comes from a single platform, Waterdrop Charity, which is one of the largest platforms in China. While it has high influence in the Chinese context, crowdfunding cultures, donor behaviors, and platform functions vary across countries and regions. Due to the difficulty of organizing platform data, we could not conduct cross-platform comparisons. Future studies could focus on institutional environmental variables and compare fundraising performance across multiple countries and platforms. Second, the textual distinctiveness variable relies on the TF-IDF metric, which captures lexical uniqueness rather than semantic subtleties or emotional tones. Future research could integrate modern natural language processing techniques to identify which narrative attributes drive donations and sharing. Regarding data use, our analysis is cross-sectional, conducted at the project’s conclusion. It does not consider the internal dynamics or the temporal progression of campaigns. Future research could employ panel data to assess the short-term effects of updates and sharing, determining optimal update frequencies based on campaign stages. Moreover, due to time constraints and the feasibility of fieldwork, this study lacks interviews with platform managers and in-depth surveys of donor behavior. Future research could conduct field studies and deep interviews to understand platform managers’ decision-making logic and donors’ behavioral motivations, providing deeper insights into the mechanisms that affect crowdfunding performance from demand and motivation perspectives.

Addressing these limitations is crucial for a more comprehensive understanding of sustainable medical crowdfunding. By solving general issues, integrating time dynamics, refining measurement methods, and ensuring causal inference, future studies can build a stronger knowledge base and inform both theory and practice.

## 8 Conclusion

This study investigates the core factors influencing internet-based medical crowdfunding and their underlying relationships through the lens of dramaturgical theory. Analyzing 8,499 projects on a major Chinese crowdfunding platform, we draw several key conclusions.

We find that frontstage performance is the visible, direct display by charitable organizations to donors, driven by both dynamic and static performance elements. Project update frequency is a key dynamic frontstage behavior, through which charities actively and frequently interact with donors by providing ongoing information. This behavior significantly enhances fundraising performance. Unlike studies that focus on superficial variables like text length or image counts, we identify textual distinctiveness and the rare-disease status of beneficiaries as static special plots, whose core logic of differentiated narrative and emotional appeal heightens the scarcity and urgency of the patient’s role, successfully mobilizing emotional resonance among donors and prompting donation decisions. Regular updates avoid donor fatigue, while differentiated storytelling taps into the unique needs of the patient, avoiding repetitive or false messaging. This combination of dynamic and static frontstage behavior not only enhances crowdfunding performance but also helps optimize the sustainable development path of medical crowdfunding from a foundational perspective.

Furthermore, backstage support capacity enhances the credibility and coherence of frontstage behavior, moderating the sustainability of performance. We find that when the initiator and executor are aligned, the backstage team coordinates better, facilitating more effective and consistent dynamic updates, which amplifies the positive effect of updates on fundraising performance. Charities with more experience, much like seasoned actors, consciously optimize their frontstage dynamic performance, leading to greater performance gains from updates.

Lastly, donor sharing behavior serves as a secondary performance, extending the frontstage performance into social networks and expanding the project’s influence. Our study finds that donor sharing acts as a mediator in the path from key independent variables to fundraising performance. Donors, no longer passive recipients, share the project based on their identification with it, essentially recreating the project’s narrative and increasing its visibility to other potential donors. This network-based interaction and diffusion continuously amplify the impact of medical crowdfunding projects, thereby improving fundraising performance.

Overall, this study provides valuable insights into the core factors and their interrelationships in internet-based medical crowdfunding, offering practical recommendations for the sustainable development of internet charity. As technology continues to evolve, its integration with philanthropy deepens, necessitating ongoing academic attention to the mechanisms of internet-based charitable fundraising and the refinement of medical crowdfunding models.

## Data Availability

The original contributions presented in the study are included in the article/supplementary material, further inquiries can be directed to the corresponding author.
